# Influence of chemotherapeutic drug-related gene polymorphisms on toxicity and survival of early breast cancer patients receiving adjuvant chemotherapy

**DOI:** 10.1186/s12885-017-3483-2

**Published:** 2017-07-26

**Authors:** Vienna Ludovini, Cinzia Antognelli, Antonio Rulli, Jennifer Foglietta, Lorenza Pistola, Rulli Eliana, Irene Floriani, Giuseppe Nocentini, Francesca Romana Tofanetti, Simonetta Piattoni, Elisa Minenza, Vincenzo Nicola Talesa, Angelo Sidoni, Maurizio Tonato, Lucio Crinò, Stefania Gori

**Affiliations:** 1 Medical Oncology Division, S. Maria della Misericordia Hospital, Azienda Ospedaliera of Perugia, Perugia, Italy; 2 Department of Experimental Medicine, University of Perugia, Piazzale Menghini 8/9, 06156 Perugia, Italy; 3 Breast Unit, Department of Surgical, University of Perugia, Perugia, Italy; 4 Oncology Department, IRCCS, Istituto di Ricerche Farmacologiche “Mario Negri”, Milan, Italy; 5 Section of Pharmacology, Department of Medicine, University of Perugia, Perugia, Italy; 6 Haematology Department, University of Perugia, Perugia, Italy; 7 Medical Oncology Division, “S. Maria” Hospital, Terni, Italy; 8 Department of Experimental Medicine, Section of Anatomic and Histology, Medical School, University of Perugia, Perugia, Italy; 9 Umbria Regional Cancer Network, Perugia, Italy; 10 Medical Oncology, Istituto Scientifico Romagnolo per lo studio e la cura dei tumori (IRST), IRCCS, Meldola, Italy; 11 Medical Oncology, SacroCuore-Don Calabria Hospital, Negrar, Verona Italy

**Keywords:** Early breast cancer, Polymorphisms, Adjuvant chemotherapy, Toxicity, Prognosis

## Abstract

**Background:**

We investigated whether *GSTT1* (“null” allele*)*, *GSTM1* (“null”allele*)*, *GSTP1* (A313G), *RFC1* (G80A), *MTHFR* (C677T), *TS* (2R/3R) polymorphisms were associated with toxicity and survival in patients with early breast cancer (EBC) treated with adjuvant chemotherapy (CT).

**Methods:**

This prospective trial included patients with stage I–III BC subjected to CT with CMF or FEC regimens. PCR-RFLP was performed for *MTHFR, RFC1* and *GSTP1*, while PCR for *TS, GSTT1* and *GSTM1* genes.

**Results:**

Among the 244 patients consecutively enrolled, 48.7% were treated with FEC and 51.3% with CMF. Patients with *TS2R/3R* genotype showed less frequently severe neutropenia (G3/G4) than those with *TS2R/2R* and *3R/3R* genotype (*p* = 0.038). Patients with *MTHFRCT* genotype had a higher probability of developing severe neutropenia than those with *MTHFR CC* genotype (*p* = 0.043). Patients with *RFC1GG* or *GSTT1-null* genotype or their combination (*GSTT1-null/RFC1GG)* were significantly associated with a shorter disease free survival (DFS) (*p* = 0.009, *p* = 0.053, *p* = 0.003, respectively) and overall survival (OS) (*p* = 0.036, *p* = 0.015, *p* = 0.005, respectively). Multivariate analysis confirmed the association of *RFC1GG* genotype with a shorter DFS (*p* = 0.018) and of *GSTT1-null* genotype of a worse OS (*p* = 0.003), as well as for the combined genotypes *GSTT1-null/RFC1GG,* (DFS: *p* = 0.004 and OS: *p* = 0.003).

**Conclusions:**

Our data suggest that *TS2R/2R* and *3R/3R* or *MTHFR CT* genotypes have a potential role in identifying patients with greater risk of toxicity to CMF/FEC and that *RFC1 GG* and *GSTT1-null* genotypes alone or in combination could be important markers in predicting clinical outcome in EBC patients.

**Electronic supplementary material:**

The online version of this article (doi:10.1186/s12885-017-3483-2) contains supplementary material, which is available to authorized users.

## Background

Breast cancer (BC) currently accounts for 20% of all female cancers worldwide and is the most frequent malignancy occurring in women [1]. There is convincing evidence that adjuvant systemic chemotherapy (AC) increases survival of patients with BC [2]. AC imparted a statistically significant reduction in the risk of BC relapse and death at 5 years of follow-up (with a hazard reduction of approximately 25%), and combination chemotherapy was found to be significantly more effective than single-agent therapy [3]. Trials included more than 15 years of follow-up and led to the conclusion that AC conferred benefit to both premenopausal and postmenopausal patients and also to node-positive and node-negative patients [4]. In general, approximately one of every four recurrences and one of seven deaths is avoided annually by adjuvant chemotherapy [5].

Among the treatments used in this adjuvant setting, the combination of cyclophosphamide (CP), methotrexate (MTX) and 5-fluorouracil (5-FU) (CMF treatment) or the combination of 5-FU, anthracycline-based chemotherapy (adriamycin or its analogue epirubicin) and CP (FAC/FEC treatment) are the most commonly used. Although the benefit of BC chemotherapy has been demonstrated, these drugs have shown the ability to induce DNA damage in eukaryotic cells [6, 7] and, consequently, chemotherapy treatment involves a risk of provoking DNAdamage even in proliferative non-cancer cells [8] therefore leading to a marked toxicity state. Adverse events represent an important physical, psychological and financial burden for the patient and society since up to 15% of the patients receiving FEC will experience at least one serious adverse event [9, 10]. Besides toxicity, another major clinical problem encountered during adjuvant CMF or FEC treatments is BC recurrence of therapeutically resistant disease and thus affecting the long-term outcome of the patient. Significant variability in drug response may occur among cancer patients treated with the same medications [11].

**Fig. 1:**
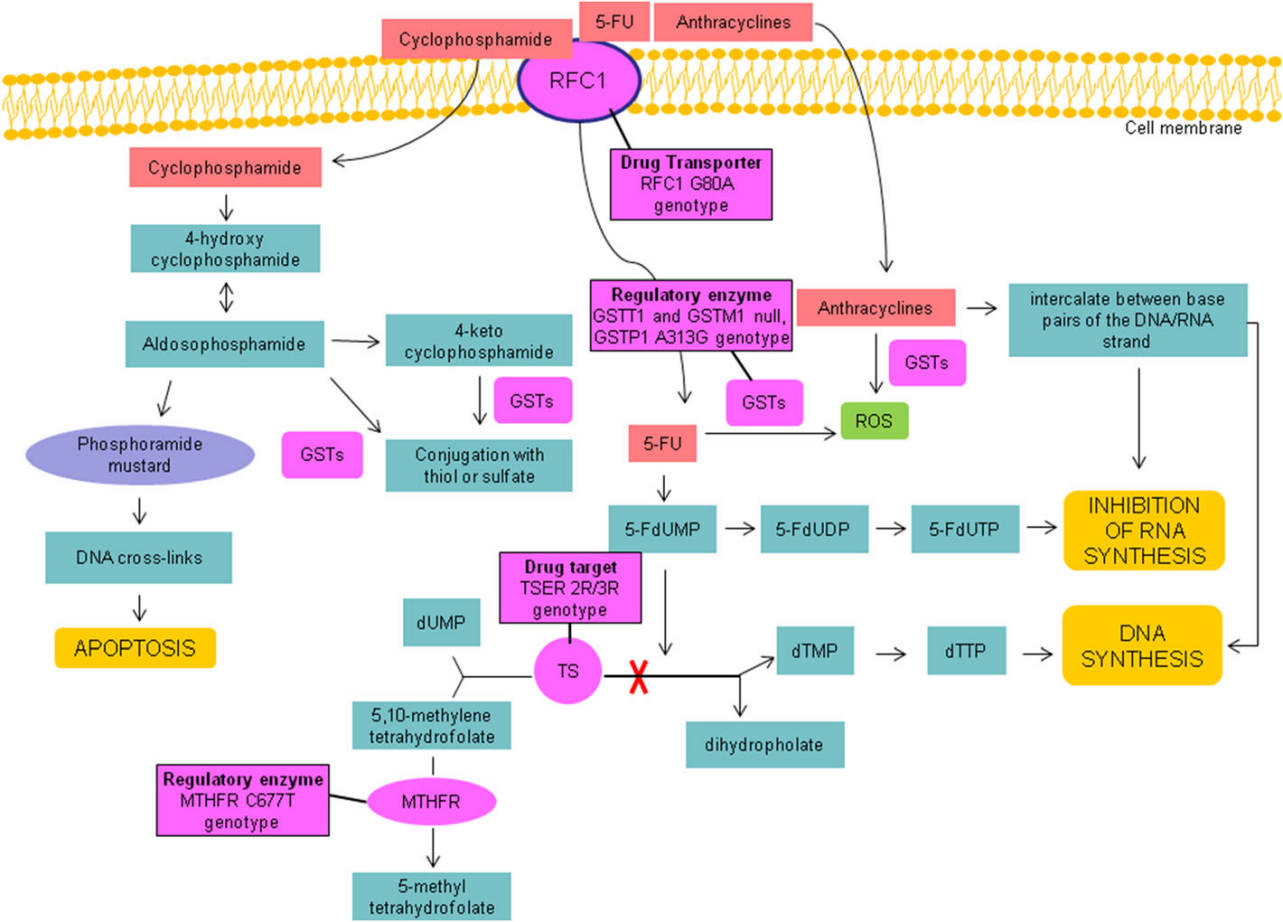
Metabolism of chemotherapeutic drugs-related gene polymorphisms. In cancer cells 5-FU is converted to 5-fluorodeoxyuridine monophosphate (5-FdUMP). 5-FdUMP inhibits the DNA synthesis by competing with deoxyuridine monophosphate (dUMP) for binding to thymidylate synthase (TS) in a complex that is stabilized by the reduced folate 5,10-methylene tetrahydrofolate. 5-FU can also inhibit RNA synthesis in a pathway that involves its metabolism to 5-fluorouridinemonophosphate (5-FUMP) and subsequent conversion to 5-fluorouridine triphosphate (5-FUTP) via 5-fluorouridine diphosphate (5-FUDP). The main effect of cyclophosphamide is due to its metabolite phosphoramide mustard that forms DNA crosslinks both between and within DNA strands at guanine N-7 positions (known as interstrand and intrastrand crosslinkages, respectively). This is irreversible and leads to cell apoptosis. Anthracyclines inhibit DNA and RNA synthesis by intercalating between base pairs of the DNA/RNA strand, thus preventing the replication of rapidly growing cancer cells. In addition, they can generate reactive oxygen species (ROS) damaging DNA, proteins and cell membranes. Glutathione S-transferases (GSTs) catalyse the detoxification of alkylating agents used in chemotherapy and/or ROS

Germline genetic variation in drug metabolizing enzymes and transporters is thought to contribute to the observed inter-individual variation in treatment toxicity and/or efficacy [12]. Recently, pharmacogenomic studies have elucidated the inherited nature of these differences in drug disposition and effects, thereby providing a stronger scientific basis for optimizing drug therapy according to each patient’s genetic constitution. Candidate genes are thymidylate synthase (*TS*), 5, 10-methylenetetrahydrofolate reductase (*MTHFR*), the reducer folate carrier (*RFC1*) and glutathione-S-transferases (*GSTs*), involved in CMF or FEC adjuvant chemotherapies transport and/or metabolism, or being targets of such drugs, as it is shown in Fig. 1. TS is an enzyme implicated in the conversion of deoxyuridine monophosphate (dUMP) into deoxythymidine monophosphate (dTMP), which is essential in DNA synthesis. The human TS gene (*hTS*) is polymorphic with either double (2R) or triple (3R) tandem repeats of a 28 base-pair sequence downstream of the cap site in the 5′ terminal regulatory region [13]. In vitro studies, the activity of a reporter gene linked to the 5′ terminal fragment of the *hTS* gene with triple (3R) tandem repeats was 2.6 times higher than that with double (2R) tandem repeats [14]. Thus, this polymorphic region *TS 2R/3R* appears to be functional and may modulate *TS* gene expression. *MTHFR* is an enzyme responsible for the metabolization of vitamin B9 (folate), which is required for DNA synthesis. A known *MTHFR* gene polymorphism consists of a 677C > T transition, in exon 4, which results in an alanine to valine substitution in the predicted catalytic domain of *MTHFR.* This substitution renders the enzyme thermolabile, and homozygotes and heterozygotes have about 70 and 35% reduced enzyme activity, respectively [15]. *RFC1* is a major MTX transporter whose impaired function has been recognized as a frequent mechanism of antifolate resistence [16]. Different gene alterations affecting *RFC1* transport properties were found in cell lines selected for antifolate resistance [17]. A polymorphism *G > A* at position 80 in exon 2 of *RFC1* gene which replaces His by Arg at position 27 of the RFC1 protein was identified. A recent study implied an effect of G > A80 in combination with C > T677 in *MTHFR* on plasma folate levels and homocysteine pools [18]. It is known that the mechanism of cytotoxicity with chemotherapy is through the generation of reactive oxygen species (ROS) and their by-products. The reactive molecules responsible for cytotoxicity of these therapies are subject to enzymatic removal, and variability of cells in sensitivity to therapy could depend, at least in part, on the availability and activity of specific metabolizing enzymes. GSTs enzymes are an important cellular defence system that protects cells from chemical injury by catalyzing conjugation of reactive electrophilic molecules with glutathione (GSH). GSTs catalyze the detoxification of some alkylating agents used in chemotherapy and detoxification of products of reactive oxidation [19]. GSTs M1 and T1 have been shown to have activity toward lipid hydroperoxides [20], and individuals lacking each of these enzymes (*null* allele) may have reduced removal of secondary organic oxidation products produced by cancer therapy and thus may have better prognoses. The pi-class human GST (GSTP1) besides playing a role in protection from oxidative damage was shown to catalyze GSH conjugation of reactive cyclophosphamide metabolites in vitro assays [21]. The present study aimed at investigating the association between *TS 2R/3R*, *MTHFR C677T*, *RFC1 G80A* and *GSTT1 null*, *GSTM1 null* or *GSTP1 A313G* polymorphisms with toxicity, disease free survival (DFS) and overall survival (OS) in Caucasian patients with early BC treated with CMF or FEC regimens.

## Methods

### Study population

This prospective study was conducted in patients with a histological diagnosis of stage I-III BC treated with conservative surgery or mastectomy, and subjected to adjuvant chemotherapy with CMF or FEC regimens. Tumor staging followed the TNM-AJCC classification [22] and the pTNM was obtained after classical pathological examination. Patients with metastatic disease and with other previous tumors were excluded from this study. Recorded clinical and pathological features for each patient included: age, menopausal status, histology, grade, stage, estrogen receptors (ER) and progesterone receptor (PgR) status, Ki67, p53, HER2 and medical adjuvant therapy. ER, PgR, Ki67, p53 and HER2 status were assessed at the time of surgery on formalin-fixed paraffin-embedded tissue blocks of the primary tumor in the Pathology Department of the University of Perugia. We used the following cut-off for considering Ki 67 positive >14%, [23] p53 positive ≥ 1%, Her2 positive IHC 3+ or IHC 2+ and FISH amplified. Written informed consent was obtained by all patients and the study was reviewed and approved by the institution’s Ethics Committee in accordance with the principles established in the Helsinki declaration.

### Chemotherapy regimen

Treatment combined regimen was as follows: CMF (cyclophosphamide 600 mg/m^2^, MTX 40 mg/m^2^ and 5-fluorouracil 600 mg/m^2^) administered on day 1 and 8 each 4 weeks, for 6 cycles; FEC (5-fluorouracil 600 mg/m^2^, 4-epirubicin 90 mg/m^2^ and cyclophosphamide 600 mg/m^2^) administered on day 1, every 21 days, for 6 cycles. Physical examination and a full blood counts were performed after each chemotherapy cycle. Hepatic and renal function tests were assessed at baseline and repeated before each cycle of treatment. All patients who had received at least one course of chemotherapy were evaluated for toxicity. Toxicity was scored every 3 weeks according to the Common Toxicity Criteria of the National Cancer Institute (NCI-CTC, version 2.0) [24].

We defined “severe toxicity” as hematological or gastrointestinal toxicity of grade 3–4.

### Genotyping analysis

Genomic DNA was extracted from 200 μL of whole blood using the Qiamp blood kit (Qiagen, Milan, Italy) according to the manufacturer’s instructions. Polymorphisms were characterized using the PCR-RFLP for genotyping analyses of *MTHFR*, *RFC1* and *GSTP1*, while PCR was used for *TS* polymorphism determination. Multiplex PCR was used to simultaneously amplify *GSTT1* and *GSTM1*, with albumin as a control gene. All primers used in this study were designed by using Primer express 2.0 software (Applied Biosystems, Italy). The primer sequences, restriction enzymes and PCR conditions used in the study are shown in Additional file 1: Table S1.

### Statistical analysis

Allele and genotype frequencies for each polymorphism were calculated and tested as to whether they were distributed according to the Hardy-Weinberg equilibrium. A chi-square test for deviation from Hardy-Weinberg equilibrium was used to estimate differences in allele frequencies. The association of each polymorphism and clinical-pathological features of the patients was assessed by means of a chi-square test. A univariate logistic regression model was used to assess the effect of the same variables, included as dummy variables on incidence of toxicity (0–1-2 grade vs. 3–4), expressing results as odds ratios (OR) and relative 95% confidence intervals (95% CIs). Disease free survival (DFS) was defined as the time from the treatment start up to the date of first progression or death from any cause, whichever came first. Patients who had not died or had disease progression at the date of analysis were censored at the last available information on status. Overall survival (OS) was defined as the time from the treatment start to the date of death from any cause. Time-to-event data were described by the Kaplan-Meier curves. Cox proportional hazards models were used for univariate and multivariate analyses to estimate and test clinical-pathological features and polymorphisms for their associations with DFS and OS. Variables statistically significant at univariate analysis (at a level of *p* < 0.10) were included in the multivariate models. Results were expressed as hazard ratio (HRs) and their 95% CIs. Due to the explorative nature of the study, no adjustment of the significance level to make allowance for multiple tests has been made. Statistical significance was set at *p* < 0.05. All statistical analyses were carried out using SAS version 9.2 (SAS Institute, Cary, NC).

## Results

### Patient characteristics

From June 2000 to September 2005 a total of 244 consecutive Caucasian patients with conservative surgery or mastectomy for primary BC, referred to the Breast Unit Surgical Department of the University of Perugia, Italy, were recruited. Histological diagnosis was confirmed by a pathologist at the Institute of Pathology, University of Perugia. The main clinical-pathological characteristics of the patients are summarized in Table 1.

**Table 1:** Baseline characteristics of patients

Characteristics	No. of patients (%)
All patients	244 (100)
Median age, years (min-max)	51.3 (26.6–75.6)
Stage	
I	111 (45.5)
II	93 (38.1)
III	40 (16.4)
Tumor size, ≤2 cm	49 (34.0)
Positive lymph nodes status	107 (43.9)
Tumor grade	
G1	18 (7.4)
G2	143 (58.6)
G3	59 (24.2)
Unknown	24 (9.8)
Histology	
Ductal infiltrating carcinoma	212 (86.9)
Other histology	32 (14.1)
Positive ER status (cut-off > 10%)	154 (63.1)
Positive PgRstatus(cut-off > 10%)	137 (56.1)
Ki67 positive status(cut-off > 14%)	112 (45.9)
Positive p53 status(cut-off ≥ 1%)	34 (13.9)
Positive HER2^a^(IHC/FISH)	26 (10.7)
Surgery	
Conservative	201 (82.4)
Mastectomy	43 (17.6)
Adjuvant chemotherapy	
CMF	124 (50.8)
FEC	120 (49.2)
Endocrine therapy	148 (60.6)
Radiotherapy	205 (84.0)

^a^IHC 3 + or IHC 2+ and FISH amplified

*ER* estrogen receptor; *PgR*, progesterone receptor

*CMF* cyclophosphamide, methotrexate, 5-fluorouracil

*FEC* 5-fluorouracil, epirubicin, cyclophosphamide

### Frequencies and associations among the polymorphisms and clinical-pathological features

The associations between genetic polymorphisms and the patient clinical-pathological features are reported in Additional file 2: Table S2.

The frequencies of genotypes *GSTT1-null* e *GSTM1-null* were 20.5% and 54.1%, respectively and *GSTM1-null* allele was significantly higher in stage I than the *GSTM1-present* allele (*p* = 0.042). The frequencies of the genotypes *GSTP1 AA, AG*, and *GG* were 59.4%, 39.3%, and 1.2%, respectively. *GSTP1 AA* genotype was significantly higher in stage III, in positive lymph nodes and in negative p53, than the *GSTP1 AG* or *GG* genotype (*p* = 0.006, *p* = 0.027 and *p* = 0.033, respectively). For *MTHFR* the frequencies of *CC, CT*, and *TT* were 27.5%, 47.5%, and 25.0%, respectively and the *MTHFR CT* or *TT* genotypes were significantly higher in stage III or in positive lymph nodes than the *MTHFR CC* genotype (*p* = 0.025 and *p* = 0.011, respectively). For the *RFC1* polymorphism, the frequencies of *GG, GA*, and *AA* were 30.3%, 46.3%, and 23.4%, respectively. The frequencies of *TS* tandem repeat genotype distribution were 32.8% in *3R3R*, 35.2% in *3R2R*, and 32.0% in *2R2R*. There was no statistically significant association among genotype distributions and tumor size, grading, ER, PgR, Ki67 and HER2 status. The genotype distribution observed was similar to that expected under Hardy-Weinberg equilibrium.

### Toxicity and effect of polymorphisms in whole BC group

All 244 patients were evaluable for toxicity. Hematological and non-hematological toxicities to CMF/FEC regimen were evaluated and are summarized in Additional file 3: Table S3. Among patients with BC who developed toxicity the prevalence of hematologic and non-hematologic toxicities of any grade was as follows: 63 neutropenia (25.8%), 58 leucopenia (23.7%), 13 anemia (5.2%), 46 mucositis (18.8%) and 35 hepatic toxicity (14.3%). Among BC patients treated with CMF (*n* = 124) the prevalence of hematologic and non-hematologic toxicities of any grade was as follows: 28 neutropenia (22.5%), 27 leucopenia (21.7%), 6 anemia (4.8%), 27 mucositis (21.7%) and 18 hepatic (14.5%) toxicity. Among BC patients treated with FEC (*n* = 120) the prevalence of hematologic and non-hematologic toxicities of any grade was as follows: 24 neutropenia (20.0%), 20 leucopenia (16.6%), 8 anemia (6.6%), 18 mucositis (15.0%) and 15 hepatic (12.5%) toxicity. There were no statistically significant differences between Table S4:CMF and FEC regimens in terms of toxicity (Additional file 3: Table S3). Grade 3/4 toxicity was observed overall in 14.3% (35/244) of patients: 10% (24/244) for hematological toxicity, 4.5% (11/244) for non-hematological toxicity (alopecia not included). A few patients experienced cycle delay (n.5 patients) or dose reduction (n.8 patients). No toxic deaths were observed in this study. Associations between genotypes and toxicities are reported in Table 2. A significant association was detected between the number of 28-bp tandem repeats in the 5′-untranslated region of the *TS* gene and the severity of toxicity. The patients with *2R/3R TS* genotype showed less frequently severe (G3/G4) neutropenia than patients with *2R/2R TS* genotype (OR = 0.25, 95% CI: 0.06–0.93p = 0.038). The patients with *CT MTHFR* genotype had a higher probability of developing severe neutropenia than patients with *CC MTHFR* genotype (OR = 8.32 95% CI: 1.06–65.2, *p* = 0.043). When considering toxicity of any grade (G1–4), patients with *2R/3R TS* genotype had a lower probability of developing oral mucositis (OR = 0.36 95% CI: 0.16–0.82, *p* = 0.015, Additional file 4: Table S4). No other statistically significant differences in toxicity were found with respect to the other polymorphisms.

**Table 2:** Association among gene polymorphisms and risk of severe toxicity (grade 3–4 vs. 0–1-2)

	HEMATOLOGIC TOXICITY								NON-HEMATOLOGIC TOXICITY							
	LEUCOPENIA				NEUTROPENIA				STOMATITIS				HEPATIC			
Genotype	0–1-2	3–4	OR (95%CI)	*p*	0–1-2	3–4	OR (95%CI)	*P*	0–1-2	3–4	OR (95%CI)	*p*	0–1-2	3–4	OR (95%CI)	*p*
GSTT1																
null	47	3	1 (reference)	0.155	45	5	1 (reference)	0.349	49	1	1 (reference)	0.822	49	1	1 (reference)	0.335
Present	190	4	0.33 (0.07–1.52)		182	12	0.59 (0.30–1.77)		191	3	0.77 (0.08–7.56)		193	1	0.25(0.02–4.13)	
GSTM1																
null	129	3	1 (reference)	0.548	122	10	1 (reference)		131	1	a		132	0	a	
Present	108	4	1.59 (0.35–7.27)		105	7	0.81 (0.30–2.21)	0.686	109	3			110	2		
GSTP1																
AA	141	4	1 (reference)	0.832	134	11	1 (reference)	0.150	142	3	1 (reference)	0.569	144	1	a	
AG	91	3	1.18 (0.26–5.39)		89	5	0.70 (0.23–2.07)		93	1	0.52 (0.05–5.04)		93	1		
GG	3	0			2	1			3	0			3	0		
RCF1																
GG	71	3	1 (reference)	0.598	69	5	1 (reference)	0.759	73	1	1 (reference)	0.824	74	0	a	
GA	110	3	0.64 (0.13–3.29)		104	9	1.19 (0.38–3.72)		111	2	1.32 (0.12–14.8)		113	0		
AA	56	1	0.42 (0.04–4.17)	0.461	54	3	0.77 (0.18–3.35)	0.724	56	1	1.30 (0.08–21.3)	0.852	55	2		
AA vs. GA + GG	0.54 (0.06–4.57)			0.571	0.69 (0.19–2.48)			0.566	1.10 (0.11–10.74)			0.938				
MTHFR																
CC	64	1	1 (reference)		66	1	1 (reference)		67	0	a		67	0	a	
CT	113	3	0.57 (0.11–2.89)	0.494	103	13	8.32 (1.06–65.2)	0.043	114	2			115	1		
TT	60	1	0.36 (0.04–3.51)	0.376	58	3	3.41 (0.35–33.7)	0.294	59	2			60	1		
TT vs. CT + CC	0.49 (0.06–4.17)			0.515	0.62 (0.17–2.25)			0.472	3.07 (0.42–22.3)			0.268				
TS-TR																
2R/2R	84	2	1 (reference)		83	3	1 (reference)		85	1	1 (reference)		86	0	a	
2R/3R	74	4	0.44 (0.08–2.47)	0.352	68	10	0.25 (0.06–0.93)	0.038	76	2	0.45 (0.04–5.03)	0.514	78	0		
3R/3R	79	1	0.23 (0.03–2.14)	0.199	76	4	0.36 (0.11–1.19)	0.095	79	1	0.48 (0.04–5.42)	0.553	78	2		
3/3R vs. 2/3R + 2/2R	0.33 (0.04–2.82)			0.313	0.61 (0.19–1.94)			0.403	0.68 (0.07–6.64)			0.740				

*OR* Odds Ratio, *CI* Confidence Intervals

^a^Due to the low number of events it was not always possible to perform the comparison test

### Survival analysis

**Fig. 2:**
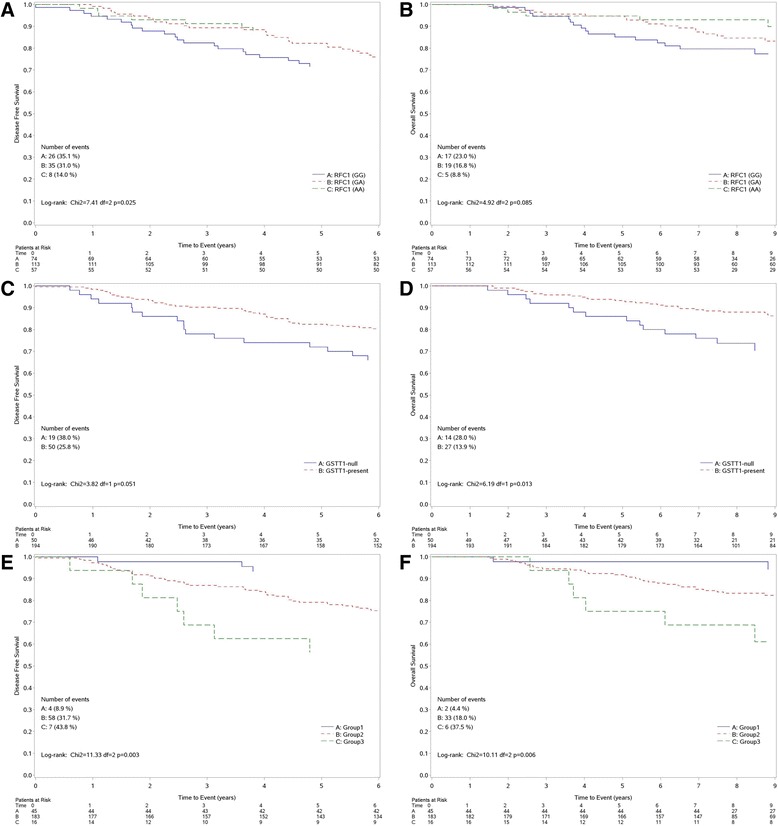
Kaplan Meier curves by *RFC1* and *GSTT1* status. Disease-Free Survival by *RFC1* polymorphism **a.** *GSTT1* status **c. **and combined genotype groups **e.** Overall Survival by *RFC1* polymorphism **b.** *GSTT1* status **d.** and combined genotype groups **f.** Combined genotype groups were as follows: group1: *GSTT1-present* and *RFC1-AA;* group2: *GSTT1-present* and *RFC1-GA/RFC1-GG* or *GSTT1-null* and *RFC1-GA/RFC1-AA;* group3: *GSTT1-null* and *RFC1-GG*

At a median follow-up of 9.2 years (interquartile range: 8.2–10.6), we observed 38 (15.6%) disease recurrences, 16 (6.6%) second tumors and 41 (16.8%) deaths. Overall the patients with recurrence and/or second tumor and/or deaths were 85 (34.8%). Loco-regional recurrence was observed in 13 patients (34.2%) and metastatic disease in 25 patients (65.8%): dominant site was visceral in 28 of 38 patients (76.7%). Results of univariate analysis for DFS and OS are reported in Table 3.Both patients with genotype *RFC1 GG* and genotype *RFC1* GA had a shorter DFS in comparison to those with genotype *AA* (HR = 2.89, 95% CI: 1.31–6.38, *p* = 0.009; HR = 2.35, 95% CI: 1.09–5.07, *p* = 0.029 for *GG* and GA, respectively (Fig. 2a- DFS curves for *RFC1*). Patients with genotype *RFC1 GG* had a shorter OS in comparison to those with genotype *AA* (HR = 2.90, 95% CI: 1.07–7.88, *p* = 0.036) while patients with genotype *RFC1 GA* did not show a different survival when compared with genotype *AA* (HR = 1.95, 95% CI: 0.79–5.22, *p* = 0.184) (Fig. 2b- OS curves for *RFC1*). DFS was also shorter in patients with genotype *GSTT1-null* when compared to patients with genotype *GSTT1-present* (HR = 1.68, 95% CI: 0.99–2.86, *p* = 0.05) (Fig. 2c- DFS curves for *GSTT1*). OS was also shorter in patients with genotype *GSTT1-null* when compared to patients with genotype *GSTT1-present* (HR = 2.22, 95% CI: 1.17–4.24, *p* = 0.015). (Fig. 2d- OS curves for *GSTT1*). The multivariate model (including age, ER/PgR positive, stage, the genotypes *GSTT1* and *RFC1*) for DFS and OS showed that the genotype *RFC1 GG* confirmed a shorter DFS when compared to *RFC1 AA* genotype (HR = 2.64, 95% CI: 1.18–5.90, *p* = 0.018), while genotype *GSTT1-null* was confirmed as a independent prognostic factor for a worse OS (HR = 2.82, 95% CI: 1.41–5.64, *p* = 0.003) (Table 4).

**Table 3:** Cox models for DFS and OS (univariate analysis)

	Univariate analysis - DFS				Univariate analysis - OS			
Variable	HR	95% CI		*p*	HR	95% CI		*p*
Age (per years)	1.01	0.99	1.04	0.270	1.05	1.01	1.08	0.005
ER- PgR-	1 (reference)				1 (reference)			
ER+ PgR- / ER- PgR+	0.72	0.40	1.30	0.273	0.64	0.30	1.40	0.269
ER+ PgR+	0.51	0.29	0.89	0.018	0.51	0.25	1.04	0.066
Stage I	1 (reference)				1 (reference)			
Stage II	2.01	1.13	3.56	0.018	3.73	1.48	9.41	0.005
Stage III	3.77	2.01	7.08	<0.001	9.77	3.85	24.82	<0.001
LN (pos vs. neg)	1.79	1.11	2.88	0.016	2.61	1.37	4.98	0.004
HER2 (pos vs. neg)	1.51	0.75	3.04	0.251	1.67	0.70	3.97	0.248
GSTT1 (null vs. present)	1.68	0.99	2.86	0.053	2.22	1.17	4.24	0.015
GSTM1 (present vs. null)	1.23	0.77	1.98	0.383	1.68	0.90	3.12	0.103
RFC1 – AA	1 (reference)				1 (reference)			
RFC1 – GA	2.35	1.09	5.07	0.029	1.95	0.73	5.22	0.184
RFC1 – GG	2.89	1.31	6.38	0.009	2.90	1.07	7.88	0.036
GSTP1 – AA	1 (reference)				1 (reference)			
GSTP1 – AG	0.77	0.46	1.26	0.297	-	-	-	0.989
GSTP1 – GG	-	-	-	0.985	0.80	0.42	1.53	0.500
MTHFR – CC	1 (reference)				1 (reference)			
MTHFR – CT	1.28	0.72	2.27	0.394	1.02	0.49	2.13	0.957
MTHFR – TT	0.85	0.42	1.71	0.642	0.96	0.41	2.25	0.920
TS-TR – 2R/2R	1 (reference)				1 (reference)			
TS-TR – 2R/3R	0.62	0.35	1.11	0.105	0.67	0.31	1.48	0.327
TS-TR – 3R/3R	0.80	0.46	1.41	0.439	1.11	0.54	2.28	0.767
Combined genotype groups*								
Group 1	1 (reference)				1 (reference)			
Group 2	4.20	1.52	11.56	0.006	4.54	1.09	18.92	0.038
Group 3	6.61	1.93	22.59	0.003	10.12	2.04	50.19	0.005

*HR* Hazard Ratio, *CI* Confidence Interval, *DFS* Disease free Survival, *OS* Overall Survival, *LN* lymph nodes

*group1: *GSTT1-present* and *RFC1-AA*

*gr*oup2: *GSTT1-present* and *RFC1-GA/RFC1-GG* or *GSTT1-null* and *RFC1-GA/RFC1-AA*

group3: *GSTT1-null* and *RFC1-GG*

**Table 4:** Cox models for DFS and OS (multivariate analysis)

	Multivariate analysis* - DFS				Multivariate analysis*– OS			
Variable	HR	95% CI		*p*	HR	95% CI		*p*
GSTT1 (nullvs. Present)	1.67	0.96	2.91	0.071	2.82	1.41	5.64	0.003
RFC1 – AA	1 (reference)				1 (reference)			
RFC1 – GA	2.15	1.00	4.65	0.051	1.53	0.57	4.14	0.402
RFC1 – GG	2.64	1.18	5.90	0.018	2.62	0.94	7.31	0.066
Combined genotype groups**								
Group 1	1 (reference)				1 (reference)			
Group 2	3.93	1.42	10.86	0.008	3.87	0.92	16.20	0.064
Group 3	6.35	1.82	22.17	0.004	11.53	2.26	58.71	0.003

*HR* Hazard Ratio, *CI* Confidence Intervals, *DFS* Disease free Survival, *OS* Overall Survival, *LN* lymph nodes

*multivariate model includes the combination of GSTT1 and RFC1genes adjusted for age, ER/PGR, stage

**group1:*GSTT1-present* and *RFC1-AA;*group2: *GSTT1-present* and *RFC1-GA/RFC1-GG* or *GSTT1-null* and *RFC1-GA/RFC1-AA*

group3: *GSTT1-null* and *RFC1-GG*

According to genotypes of *GSTT1* and *RFC1* genes we classified patients in three groups: the first with *GSTT1-present* and *RFC1-AA* (group1), the second with *GSTT1-present* and *RFC1-GA/RFC1-GG* or *GSTT1-null* and *RFC1-GA/RFC1-AA* (group2)*,* and the third with *GSTT1-null* and *RFC1-GG* (group3)*.*

Kaplan-Meier curves for DFS and OS are reported in Fig. 2e and f, respectively. At univariate analysis, confirmed at multivariate analysis (Table 4) both for DFS and OS, group2 showed a worse prognosis compared with group1 (HR = 4.20, 95% CI 1.52–11.56, *P* = 0.006; HR = 4.54, 95% CI 1.09–18.92, *P* = 0.038 for DFS and OS respectively). A greater difference was detected when compared group3 with group1 (HR = 6.61, 95% CI 1.93–22.59, *P* = 0.003; HR = 10.12, 95% CI 2.04–50.19, *P* = 0.005 for DFS and OS respectively).

## Discussion

In the present study, we demonstrated that among BC patients who received CMF or FEC, those possessing the *TS 2R/3R* variant showed a significantly lower risk of severe toxicity (grade 3–4) for neutropenia and, when considering toxicity of any grade (G1–4), the same variant conferred a lower probability of developing oral mucositis. Our data are in agreement with previously published studies [25–27] confirming a significant inverse association of *TS 2R/3R* polymorphism and severity toxicity. However, whereas in the study by Lecomte et al. patients with the *2R/2R* genotype were 20 times more likely to have severe toxicity compared with *3R/3R* carriers, this effect was much less pronounced in our study and more similar to the results of Schwab’s study [28]. However, the role of other 5-FU catabolism-involved polymorphisms, such as dihydropyrimidine dehydrogenase (DPYD), should be explored to improve prediction of 5-FU toxicity [29]. At present, the real predictive value of *MTHFR C677T* polymorphism on MTX and 5-FU toxicity is not completely established. In our study, we found that the patients with *MTHFR CT* genotype had a higher probability of developing severe neutropenia than patients with *MTHFR CC* genotype. Some recent studies have shown increased toxicity in 677 T–carriers treated with methotrexate [30–32], although other studies did not confirm such an association [33, 34]. Different methotrexate doses and schemes as well as diverse nutritional/folate status might account, at least in part, for these discrepant results. Probably, the heterozygous effects of *MTHFR CT* and *TS 2R/3R* genotypes as compared to each homozygous effect might be justified by considering that exogen factors, environmental conditions, dietary habits and lifestyle might play an important role [25–27, 35, 36]. No other significant differences in toxicity were found with respect to the other polymorphisms. There are a few studies on the role of GSTs isoenzymes on mortality in BC survivors drawn from community practice. The majority of these studies have small sample sizes, are based on participants diagnosed prior to 1999 and on women undergoing chemotherapy and/or radiotherapy. In addition, most of them examined only one GST gene (usually GSTP1). In our study, we showed that genotype *GSTT1-null* was associated with worse DFS and OS in EBC patients. This association was maintained in the multivariate model only for OS independently of age and other traditional predictors of prognosis. Our results are based on the assumption that the individuals with *GSTT1-null* genotype, that is associated with an absence of enzyme activity, are considered to be at increased risk for malignancies due to reduced efficiency in protection against environmental carcinogens [37, 38]. Conversely, Ambrosone et al. [39], showed that *GSTM1-null* and *GSTT1-null* genotypes predicted significantly better DFS and OS, both individually or in combination. Our results on *GSTM1*genotype are in agreement with those of Lizard-Nacol et al. [40] who, showed no effect of *GSTM1-null* genotype on DFS or OS among 92 women with advanced BC who had received cyclophosphamide, doxorubicin, and 5-FU. Whereas, Kristensen et al. [41] found that patients with *GSTM1-null* allele had a significantly shorter OS. Moreover, Yu Ke-Da et al. [42] showed a more complicated role for *GSTM1* that should be considered in breast cancer risk prediction. The results of this study indicated a U-shaped association of *GSTM1* with breast cancer, which challenges the linear gene-dosage effect of *GSTM1* that was previously proposed. This effect was due to a new SNP, rs412543 (−498C > G) located in the promoter region that decreased gene transcription by 30–40% via reducing the DNA-binding affinity of AP-2. In contrast to these previous studies, our study is the only one to examine adjuvant therapy in a population of patients with a relatively uniform recurrence risk, with a longer follow-up (9.2 years), providing a homogeneous patient population in which to study treatment related genotypes and outcomes. Genetic background differences among races account for differences in the frequencies of allelic variants so that the association of polymorphic variants with a disease risk can significantly vary among populations. As far as we know, scanty information is available on the association of chemotherapeutic drug-related gene polymorphisms on toxicity and survival of breast cancer patients in non Caucasian populations. The results of Yang et al. showed no association between any of the GSTM1 or GSTT1 genotypes in patients with breast carcinoma who were treated with chemotherapy [43].

*RFC1* genotypes, as predictors of BC treatment efficacy, have not been previously reported. Recent evidence suggests that *G80A* polymorphism in *RFC1* is associated with altered folate/antifolate levels and may influence the efficacy of therapy with MTX [39]. Data suggest that subjects carrying the homozygous mutant *AA* genotype tend to have higher plasma folate and MTX levels and higher erythrocyte polyglutamate levels compared with those with the wild type or heterozygous genotype. In our study, for the first time to our knowledge, we showed that patients with *RFC1 GG* genotype had a shorter DFS and OS than carriers of the *AA* genotype. These observations are in keeping with previous studies on rheumatoid arthritis (RA). The work of Drozdzik et al. [44] showed that patients with *RFC1 AA* genotype responded to the therapy more effectively than carriers of *AG* and *GG* genotypes. The remission of RA symptoms was significantly higher (3.32-fold) in *AA* carriers in comparison to *GG* individuals. In contrast to RA patients, the study on acute lymphoblastic leukemia of Laverdiere et al. [45] showed children with *AA* genotype had worse prognoses than patients with *GG* genotype, and *AA* genotype was associated with higher plasma levels of MTX than other genotypes. Moreover, we showed, in an explorative analysis, that the combined genotypes (*GSTT1-null/RFC1-GG)* had a negative prognostic effect on DFS and OS. This subgroup of tumors could have a more aggressive clinical course and the availability of a non-invasive, repeatable and reproducible technique to detect polymorphisms in the blood appears to be a useful tool for identifying high-risk BC patients. Therefore, further large sample size and well designed studies are greatly needed to confirm these preliminary results. Limitations of our study include relatively small sample size and low number of events, thus we were not able to evaluate the association with outcome by subgroups, such as menopausal status. Nevertheless, the association between GST polymorphisms and BC survival, showed by our results seems to be in agreement with those of the literature [39, 40].

The cohort was established before some current treatments, such as aromatase inhibitors, and Her2/neu targeted therapies were available. Therefore, we cannot estimate what associations GST isoenzymes might have with survival in women using these treatments. However, our study has a larger sample size than most prior studies examining the association between *GST* polymorphisms and survival and it is the first study to evaluate *RFC1* genotypes as predictors of BC treatment efficacy.

## Conclusions

In conclusion, our study provides important novel information about the potential role of drug-transporter enzyme polymorphisms in the outcome after adjuvant therapy for EBC. Confirmation of these findings in a large sample size and well designed studies and supportive mechanistic data will ultimately allow the potential for drug-transporter genotyping to be realized in the clinic to individualize and optimize EBC therapy.

## Additional files

Additional file 1: Table S1. Characteristics of the studied polymorphisms. (DOC 46 kb)

Additional file 2: Table S2. Association among gene polymorphisms and clinical-pathological features. (DOC 99 kb)

Additional file 3:Table S3. CMF/FEC treatment-related toxicity graded according to the NCI- CTC v.2.0. (DOC 55 kb)

Additional file 4: Table S4. Association among gene polymorphisms and risk of toxicity of any grade (grade1–2–3-4 vs 0). (DOC 83 kb)

## References

[1] Parkin DM, Bray F, Ferlay J, Pisani P (2005). Global cancer statistics, 2002. CA Cancer J Clin.

[2] Guarneri V, Conte PF (2004). The curability of breast cancer and the treatment of advanced disease. Eur J Nucl Med Mol Imaging.

[3] The Ludwig Breast Cancer Study Group (1988). Combination adjuvant chemotherapy for node-positive breast cancer inadequacy of a single erioperative cycle. N Engl J Med.

[4] Early Breast Cancer Trialists’ Collaborative Group (1992). Systemic treatment of early breast cancer by hormonal cytotoxic or immune therapy 133 randomised trials involving 31000 recurrences and 24000 deaths among 75000 women. Lancet.

[5] Early Breast Cancer Trialists’ Collaborative Group (1998). Polychemotherapy for early breast cancer: an overview of the randomised trials. Lancet.

[6] Anderson D, Bishop JB, Colin Garner R, Ostrosky-Wegman P, Selby PB (1995). Cyclophosphamide: review of its mutagenicity for an assessment of potential germ cell risks. Mutat Res.

[7] Longley DB, Harkin DP, Johnston PG (2003). 5-fluorouracil: mechanisms of action and clinical strategies. Nat Rev Cancer.

[8] Kopjar N, Garaj-Vrhovac V, Milas I (2002). Assessment of chemotherapy-induced DNA damage in peripheral blood leukocytes of cancer patients using the alkaline comet assay. Teratog Carcinog Mutagen.

[9] Roché H, Fumoleau P, Spielmann M, Canon JL, Delozier T, Serin D, Symann M, Kerbrat P, Soulié P, Eichler F, Viens P, Monnier A, Vindevoghel A, Campone M, Goudier MJ, Bonneterre J, Ferrero JM, Martin AL, Genève J, Asselain B (2006). Sequential adjuvant epirubicin-based and docetaxel chemotherapy for node-positive breast cancer patients the FNCLCC PACS 01. Trial J Clin Oncol.

[10] Hasset MJ, O’Malley AJ, Pakes JR, Newhouse JP, Earle CC (2006). Frequency and cost of chemotherapyrelated serious adverse effects in a population sample of women with breast cancer. J Natl Cancer Inst.

[11] Choi JY, Nowell SA, Blanco JG, Ambrosone CB (2006). The role of genetic variability in drug metabolism pathways in breast cancer prognosis. Pharmacogenomics.

[12] Gonzalez-Neira A (2012). Pharmacogenetics of chemotherapy efficacy in breast cancer. Pharmacogenomics.

[13] Horie N, Aiba H, Ogura K, Hojo H, Takeishi K (1995). Functional analysis and DNA polymorphism of the tandemly repeated sequences in the 59-terminal regulatory region of the human gene for thymidylate synthase. Cell Struct Funct.

[14] Horie N, Chimoto M, Nozawa R, Takeishi K (1993). Characterization of the the Pharmacogenomics journal regulatory sequences and nuclear factors that function in cooperation with the promoter of the human thymidylate synthase gene. Biochem Biophys Acta.

[15] Frost P, Blom HJ, Milos R, Goyette P, Sheppard CA, Matthews RG, Boers GJ, den Heijer M, Kluijtmans LA, van den Heuvel LP, Rozen R (1995). A candidate genetic risk factor for vascular disease: a common mutation in methylenetetrahydrofolatereductase. Nat Genetics.

[16] Gorlick R, Goker E, Trippett T, Waltham M, Banerjee D, Bertino JR (1996). Intrinsic and acquired resistance to methotrexate in acute leukemia. N Engl J Med.

[17] Moskow JA (1997). Reduced folate carrier gene (RFC1) expression and anti-folate resistance in transfected and non-selected cell lines. Int J Cancer.

[18] Chango A, Emery-Fillon N, de Courcy GP, Lambert D, Pfister M, Rosenblatt DS, Nicolas JP (2000). A polymorphism (80G/a) in the reduced folate carrier gene and its associations with folate status and homocysteinemia. Mol Genet Metab.

[19] Hayes JD, Pulford DJ (1995). The glutathione S-transferase supergene family: regulation of GST and the contribution of the isoenzymes to cancer chemoprotection and drug resistance. Crit Rev Biochem Mol Biol.

[20] Hurst R, Bao Y, Jemth P, Mannervik B, Williamson G (1998). Phospholipid hydroperoxide glutathione peroxidase activity of human glutathione transferases. Biochem J.

[21] Dirven HA, Van Ommen B, Van Bladeren PJ (1994). Involvement of human glutathione S-transferaseisoenzymes in the conjugation of cyclophosphamide metabolites with glutathione. Cancer Res.

[22] Singletary SE, Allred C, Ashley P, Bassett LW, Berry D, Bland KI, Borgen PI, Clark G, Edge SB, Hayes DF, Hughes LL, Hutter RV, Morrow M, Page DL, Recht A, Theriault RL, Thor A, Weaver DL, Wieand HS, Greene FL (2002). Revision of the American joint committee on cancer staging system for breast cancer. J Clin Oncol.

[23] Goldhirsch A, Wood WC, Coates AS, Gelber RD, Thürlimann B, Senn HJ (2011). Panel members. Strategies for subtypes--dealing with the diversity of breast cancer: highlights of the St. Gallen international expert consensus on the primary therapy of early breast cancer 2011. Ann Oncol.

[24] Common Terminology Criteria for Adverse Events v2.0 (CTCAE) Available at: ctepcancergov/reporting/ctchtml [Last Accessed 1 January 2010].

[25] Pullarkat ST, Stoehlmacher J, Ghaderi V, Xiong YP, Ingles SA, Sherrod A, Warren R, Tsao-Wei D, Groshen S, Lenz HJ (2001). Thymidylate synthase gene polymorphism determines response and toxicity of 5-FU chemotherapy. Pharmacogenomics J.

[26] Adleff V, Hitre E, Köves I, Orosz Z, Hajnal A, Kralovánszky J (2004). Heterozygote deficiency in thymidylate synthase enhancer region polymorphism genotype distribution in Hungarian colorectal cancer patients. Int J Cancer.

[27] Lecomte T, Ferraz JM, Zinzindohoué F, Loriot MA, Tregouet DA, Landi B, Berger A, Cugnenc PH, Jian R, Beaune P, Laurent-Puig P (2004). Thymidylate synthase gene polymorphism predicts toxicity in colorectal cancer patients receiving 5-fluorouracil-based chemotherapy. Clin Cancer Res.

[28] Schwab M, Zanger UM, Marx C, Schaeffeler E, Klein K, Dippon J, Kerb R, Blievernicht J, Fischer J, Hofmann U, Bokemeyer C, Eichelbaum M (2008). German 5-FU toxicity study group: role of genetic and nongenetic factors for fluorouracil treatment-related severe toxicity: a prospective clinical trial by the German 5-FU toxicity study group. JCO.

[29] Van Kuilenburg AB (2004). Dihydropyrimidine dehydrogenase and the efficacy and toxicity of 5-fluorouracil. Eur J Cancer.

[30] Ulrich CM, Yasui Y, Storb R, Schubert MM, Wagner JL, Bigler J, Ariail KS, Keener CL, Li S, Liu H, Farin FM, Potter JD (2001). Pharmacogenetics of methotrexate toxicity among marrow transplantation patients varies with the methylenetetrahydrofolatereductase C677T polymorphism. Blood.

[31] Chiusolo P, Reddiconto G, Casorelli I, Laurenti L, Sorà F, Mele L, Annino L, Leone G, Sica S (2002). Preponderance of methylenetetrahydrofolatereductase C677T homozygosity among leukemia patients intolerant to methotrexate. Ann Oncol.

[32] Toffoli G, Russo A, Innocenti F, Corona G, Tumolo S, Sartor F, Mini E, Baiocchi M (2003). Effect of methylenetetrahydrofolatereductase 677C→T polymorphism on toxicity and homocysteine plasma level after chronic methotrexate treatment of ovarian cancer patients. Int J Cancer.

[33] Kishi S, Griener J, Cheng C, Das S, Cook EH, Pei D, Hudson M, Rubnitz J, Sandlund JT, Pui CH, Relling MV (2003). Homocysteinepharmacogenetics and neurotoxicity in children with leukemia. J Clin Oncol.

[34] Seidemann K, Book M, Zimmermann M, Meyer U, Welte K, Stanulla M, Reiter A (2006). MTHFR 677 (C→T) polymorphism is not relevant for prognosis or therapy-associated toxicity in pediatric NHL: results from 484 patients of multicenter trial NHL-BFM 95. Ann Hematol.

[35] Sidoti A, Antognelli C, Rinaldi C, D'Angelo R, Dattola V, Girlanda P, Talesa V, Amato A (2007). Glyoxalase I A111E, paraoxonase 1 Q192R and L55M polymorphisms: susceptibility factors of multiple sclerosis?. Mult Scler.

[36] Antognelli C, Del Buono C, Ludovini V, Gori S, Talesa VN, Crinò L, Barberini F, Rulli A (2009). CYP17, GSTP1, PON1 and GLO1 gene polymorphisms as risk factors for breast cancer: an Italian case-control study. BMC Cancer.

[37] McIlwain CC, Townsend DM, Tew KD (2006). Glutathione S-transferasepolymorphisms: cancer incidence and therapy. Oncogene.

[38] Gao LB, Pan XM, Li LJ, Liang WB, Bai P, Rao L, Su XW, Wang T, Zhou B, Wei YG, Zhang L (2001). Null genotypes of GSTM1 and GSTT1 contribute to risk of cervical neoplasia: an evidence-based meta-analysis. PLoS One.

[39] Ambrosone CB, Sweeney C, Coles BF, Thompson PA, McClure GY, Korourian S, Fares MY, Stone A, Kadlubar FF, Hutchins LF (2001). Polymorphisms in glutathione S-transferases (GSTM1 and GSTT1) and survival after treatment for breast cancer. Cancer Res.

[40] Lizard-Nacol S, Coudert B, Colosetti P, Riedinger JM, Fargeot P, Brunet-Lecomte P (1999). Glutathione S-transferase M1 null genotype: lack of association with tumour characteristics and survival in advanced breast cancer. Breast Cancer Res.

[41] Nedelcheva Kristensen V, Andersen TI, Erikstein B, Geitvik G, Skovlund E, Nesland JM, Børresen-Dale AL (1998). Single tube multiplex polymer-ase chain reaction genotype analysis of GSTM1 GSTT1 and GSTP1: relation of genotypes to TP53 tumor status and clinicopathological variables in breast cancer patients. Pharmacogenetics.

[42] Yu KD, Di GH, Fan L, Wu J, Hu Z, Shen ZZ, Huang W, Shao ZM (2009). A functional polymorphism in the promoter region of GSTM1 implies a complex role for GSTM1 in breast cancer. FASEB J.

[43] Yang G, Shu XO, Ruan ZX, Cai QY, Jin F, Gao YT, Zheng W (2005). Genetic polymorphisms in glutathione-S-Transferase genes (GSTM1, GSTT1, GSTP1) and survival after chemotherapy for invasive breast carcinoma. Cancer.

[44] Drozdzik M, Rudas T, Pawlik A, Gornik W, Kurzawski M, Herczynska M (2007). Reduced folate carrier-1 80G>A polymorphism Yu KD, Di GH, Fan L, Wu J, Hu Z, Shen ZZ, Huang W, Shao ZM. A functional polymorphism in the promoter region of GSTM1 implies a complex role for GSTM1 in breast cancer. FASEB J. 2009 Jul;23(7):2274–87affects methotrexate treatment outcome in rheumatoid arthritis. Pharmacogenomics J.

[45] Laverdiere C, Chiasson S, Costea I, Moghrabi A, Krajinovic M (2002). Polymorphism G80A in the reduced folate carrier gene and its relationship to methotrexate plasma levels and outcome of childhood acute lymphoblastic leukemia. Blood.

