# Supplementary material for: Influence of chemotherapeutic drug-related gene polymorphisms on toxicity and survival of early breast cancer patients receiving adjuvant chemotherapy
Source: BMC Cancer. 2017 Jul 26;17:502. doi: 10.1186/s12885-017-3483-2 (PMC5530465; doi:10.1186/s12885-017-3483-2)
Supplement: Supplementary file 1 — Characteristics of the studied polymorphisms. (DOC 46 kb) [file 12885_2017_3483_MOESM1_ESM.doc]

**Table S1.** Characteristics of the studied polymorphisms.

| **Gene polymorphism** | **Primers** | **PCR size** | **PCR condition*** | **Restriction enzyme** |
| --- | --- | --- | --- | --- |
| GSTT1 | [F]:5’-TTCCTTACTGGTCCTCACATCTC-3’  [R]:5’-TCACCGGATCATGGCCAGCA-3’ | 350 | 95°C, 30 sec  64°C, 60 sec x 30 cycles  72°C, 60 sec | _ |
| GSTM1 | [F]:5’-GAACTCCCTGAAAAGCTAAAGC-3’  [R]:5’-GTTGGGCTCAAATATACGGTGG-3’ | 215 | 95°C, 30 sec  64°C, 60 sec x 30 cycles  72°C, 60 sec | _ |
| GSTP1 Ile105Val (A313G) | [F]:5’-GTGGCTCCTGCGTTTCCCCC-3’  [R]:5’-CTCCGAGCCGGCCACAGGCAT-3’ | 433 | 95°C, 30 sec  62°C, 10 sec x 35 cycles  72°C, 25 sec | *BsmAI* |
| RFC1  Arg27His (G80A) | [F]:5’-AGTGTCACCTTCGTCCCCTC-3’  [R]:5’-CTCCCGCGTGAAGTTCTTGT-3’ | 230 | 95°C, 30 sec  58°C, 30 sec x 35 cycles  72°C, 45 sec | *Cof*I |
| MTHFR Ala222Val  (C677T) | [F]:5’-TGAAGGAGAAGGTGTCTGCGGGA-3’  [R]:5’-AGGACGGTGCGGTGAGAGTG-3’ | 400 | 95°C, 30 sec  55°C, 30 sec x 35 cycles  72°C, 60 sec | *Hinf*I |
| TS 28bp  tandem repeat | [F]:5’-GTGGCTCCTGCGTTTCCCCC-3’  [R]:5’-GCTCCGAGCCGGCCACAGGCATGGCGCGG-3’ | 240 | 95°C, 30 sec  62°C, 10 sec x 35 cycles  72°C, 25 sec: | _ |

*denaturation was performed at 95° C for 5 minutes and PCR reaction volume was 25 µl for all genes.

PCR fragments were separated on a 2.5-3.0% agarose gel and visualized after staining with ethidium bromide.
