# Supplementary material for: Influence of chemotherapeutic drug-related gene polymorphisms on toxicity and survival of early breast cancer patients receiving adjuvant chemotherapy
Source: BMC Cancer. 2017 Jul 26;17:502. doi: 10.1186/s12885-017-3483-2 (PMC5530465; doi:10.1186/s12885-017-3483-2)
Supplement: Supplementary file 2 — Association among gene polymorphisms and clinical-pathological features. (DOC 99 kb) [file 12885_2017_3483_MOESM2_ESM.doc]

**Table S2 –** Association among gene polymorphisms and clinical-pathological features

|  | **GSTT1** | | | **GSTM1** | | | **GSTP1** | | | | **RCF1** | | | | **MTHFR** | | | | **TS-TR** | | | |
| --- | --- | --- | --- | --- | --- | --- | --- | --- | --- | --- | --- | --- | --- | --- | --- | --- | --- | --- | --- | --- | --- | --- |
| **Characteristic** | **null** | **present** | **p** | **null** | **present** | **p** | **AA** | **AG** | **GG** | **p** | **GG** | **GA** | **AA** | **p** | **CC** | **CT** | **TT** | **p** | **2R/2R** | **2R/3R** | **3R/3R** | **p** |
| **Stage** |  |  |  |  |  |  |  |  |  |  |  |  |  |  |  |  |  |  |  |  |  |  |
| I | 24 | 87 |  | 65 | 46 |  | 55 | 53 | 3 |  | 35 | 49 | 27 |  | 41 | 20 | 6 |  | 34 | 38 | 39 |  |
| II | 18 | 75 |  | 52 | 41 |  | 58 | 33 | 0 |  | 28 | 44 | 21 |  | 20 | 47 | 20 |  | 29 | 35 | 29 |  |
| III | 8 | 32 | 0.753 | 15 | 25 | 0.042 | 32 | 8 | 0 | 0.006 | 11 | 20 | 9 | 0.974 | 6 | 26 | 14 | 0.025 | 15 | 13 | 12 | 0.897 |
| **Tumor size**, |  |  |  |  |  |  |  |  |  |  |  |  |  |  |  |  |  |  |  |  |  |  |
| ≤ 2 cm | 36 | 140 |  | 99 | 77 |  | 100 | 72 | 3 |  | 50 | 86 | 40 |  | 50 | 85 | 41 |  | 51 | 63 | 62 |  |
| >2 cm | 13 | 51 | 0.981 | 30 | 34 | 0.199 | 44 | 19 | 0 | 0.153 | 23 | 26 | 15 | 0.458 | 15 | 29 | 20 | 0.432 | 27 | 20 | 17 | 0.145 |
| **Lymph node status** |  |  |  |  |  |  |  |  |  |  |  |  |  |  |  |  |  |  |  |  |  |  |
| Negative | 30 | 107 |  | 81 | 56 |  | 73 | 61 | 3 |  | 34 | 57 | 46 |  | 48 | 58 | 31 |  | 44 | 48 | 45 |  |
| Positive | 20 | 87 | 0.539 | 51 | 56 | 0.075 | 72 | 33 | 0 | 0.027 | 23 | 56 | 28 | 0.240 | 19 | 58 | 30 | 0.011 | 42 | 30 | 35 | 0.410 |
| **Tumor grade** |  |  |  |  |  |  |  |  |  |  |  |  |  |  |  |  |  |  |  |  |  |  |
| G1 | 5 | 13 |  | 12 | 6 |  | 11 | 6 | 0 |  | 5 | 11 | 2 |  | 5 | 9 | 4 |  | 5 | 7 | 7 |  |
| G2 | 27 | 116 |  | 80 | 63 |  | 80 | 59 | 3 |  | 43 | 68 | 32 |  | 40 | 71 | 32 |  | 45 | 45 | 45 |  |
| G3 | 12 | 47 | 0.719 | 26 | 33 | 0.054 | 41 | 18 | 0 | 0.379 | 20 | 23 | 16 | 0.493 | 14 | 23 | 22 | 0.290 | 23 | 23 | 24 | 0.230 |
| **ER** ^a^ |  |  |  |  |  |  |  |  |  |  |  |  |  |  |  |  |  |  |  |  |  |  |
| Negative | 22 | 28 |  | 55 | 77 |  | 55 | 35 | 0 |  | 27 | 44 | 19 |  | 21 | 43 | 26 |  | 31 | 30 | 29 |  |
| Positive | 68 | 126 | 0.243 | 35 | 77 | 0.093 | 90 | 59 | 3 | 0.406 | 47 | 69 | 38 | 0.772 | 46 | 73 | 35 | 0.419 | 55 | 48 | 51 | 0.940 |
| **PgR** ^a^ |  |  |  |  |  |  |  |  |  |  |  |  |  |  |  |  |  |  |  |  |  |  |
| Negative | 27 | 80 |  | 56 | 51 |  | 65 | 41 | 1 |  | 33 | 53 | 21 |  | 27 | 52 | 28 |  | 32 | 38 | 37 |  |
| Positive | 23 | 114 | 0.106 | 76 | 61 | 0.626 | 80 | 53 | 2 | 0.914 | 41 | 60 | 36 | 0.455 | 40 | 64 | 33 | 0.782 | 46 | 48 | 47 | 0.802 |
| **Ki67** ^b^ |  |  |  |  |  |  |  |  |  |  |  |  |  |  |  |  |  |  |  |  |  |  |
| Negative | 24 | 88 |  | 62 | 50 |  | 64 | 46 | 2 |  | 29 | 54 | 29 |  | 30 | 52 | 30 |  | 39 | 33 | 40 |  |
| Positive | 26 | 106 | 0.739 | 70 | 62 | 0.717 | 81 | 48 | 1 | 0.597 | 45 | 59 | 28 | 0.356 | 37 | 64 | 31 | 0.839 | 39 | 53 | 40 | 0.221 |
| **p53** ^c^ |  |  |  |  |  |  |  |  |  |  |  |  |  |  |  |  |  |  |  |  |  |  |
| Negative | 39 | 171 |  | 113 | 97 |  | 131 | 74 | 3 |  | 66 | 99 | 45 |  | 57 | 102 | 51 |  | 63 | 77 | 70 |  |
| Positive | 11 | 23 | 0.065 | 19 | 15 | 0.822 | 14 | 20 | 0 | 0.033 | 8 | 14 | 12 | 0.200 | 10 | 14 | 10 | 0.706 | 15 | 9 | 10 | 0.245 |
| **HER2^d^** |  |  |  |  |  |  |  |  |  |  |  |  |  |  |  |  |  |  |  |  |  |  |
| Negative | 44 | 174 |  | 116 | 102 |  | 130 | 83 | 3 |  | 62 | 103 | 53 |  | 60 | 105 | 53 |  | 69 | 77 | 72 |  |
| Positive | 6 | 20 | 0.730 | 16 | 10 | 0.422 | 15 | 11 | 0 | 0.789 | 12 | 10 | 4 | 0.168 | 7 | 11 | 8 | 0.757 | 9 | 9 | 8 | 0.950 |

^a^ (cut-off>10%),^b^(cut-off>14%), ^c^ (cut-off≥1%),^d^ IHC3+ or IHC 2+ and FISH amplification

CMF: cyclophosphamide, methotrexate, 5-fluorouracil

FEC: 5-fluorouracil, epirubicin, cyclophosphamide
