# Supplementary material for: Influence of chemotherapeutic drug-related gene polymorphisms on toxicity and survival of early breast cancer patients receiving adjuvant chemotherapy
Source: BMC Cancer. 2017 Jul 26;17:502. doi: 10.1186/s12885-017-3483-2 (PMC5530465; doi:10.1186/s12885-017-3483-2)
Supplement: Supplementary file 3 — CMF/FEC treatment-related toxicity graded according to the NCI- CTC v.2.0. (DOC 55 kb) [file 12885_2017_3483_MOESM3_ESM.doc]

**Table S3.** CMF/FEC treatment-related toxicity, graded according to the NCI- CTC v.2.0

|  | **All patients (N=244)** | | | | | **CMF (N=124)** | | | | | **FEC (N=120)** | | | | |  |
| --- | --- | --- | --- | --- | --- | --- | --- | --- | --- | --- | --- | --- | --- | --- | --- | --- |
|  | Grade | | | | | Grade | | | | | Grade | | | | |  |
| Toxicity – N (%) | 0 | I | II | III | IV | 0 | I | II | III | IV | 0 | I | II | III | IV | p* |
| **Hematologic** |  |  |  |  |  |  |  |  |  |  |  |  |  |  |  |  |
| Anemia | 230 (94) | 11 (5) | 3 (1) | - (0) | - (0) | 118 (95) | 5 (4) | 1 (1) | - (0) | - (0) | 112 (93) | 6 (5) | 2 (2) | - (0) | - (0) | 0.484 |
| Leucopenia | 186 (76) | 20 (8) | 31 (13) | 7 (3) | - (0) | 97 (78) | 11 (9) | 14 (11) | 2 (2) | - (0) | 89 (74) | 9 (8) | 17 (14) | 5 (4) | - (0) | 0.252 |
| Neutropenia | 181 (74) | 29 (12) | 17 (7) | 12 (5) | 5 (2) | 96 (77) | 14 (11) | 9 (7) | 4 (3) | 1 (1) | 85 (71) | 15 (13) | 8 (7) | 8 (7) | 4 (3) | 0.098 |
| **Non-hematologic** |  |  |  |  |  |  |  |  |  |  |  |  |  |  |  |  |
| Mucositis | 198 (81) | 25 (10) | 17 (7) | 4 (2) | - (0) | 97 (78) | 15 (12) | 10 (8) | 2 (2) | - (0) | 101(84) | 10 (8) | 7 (6) | 2 (2) | - (0) | 0.346 |
| Hepatic | 209 (86) | 24 (10) | 9 (4) | 2 (1) | - (0) | 106 (85) | 14 (11) | 3 (2) | 1 (1) | - (0) | 102 (87) | 9 (8) | 5 (4) | 1 (1) | - (0) | 0.737 |
| Cardiac | 235 (96) | 3 (1) | 5 (2) | 1 (0) | - (0) | 118 (95) | 2 (2) | 4 (3) | - (0) | - (0) | 117 (98) | 1 (1) | 1 (1) | 1 (1) | 1 (1) | 0.503 |
| Diarrhea | 231 (95) | 8 (3) | 5 (2) | - (0) | - (0) | 118 (95) | 4 (3) | 2 (2) | - (0) | - (0) | 113 (94) | 4 (3) | 3 (3) | - (0) | - (0) | 0.657 |
|  |  |  |  |  |  |  |  |  |  |  |  |  |  |  |  |  |

CMF: cyclophosphamide, methotrexate, 5-fluorouracil

FEC: 5-fluorouracil, epirubicin, cyclophosphamide

*p: comparison between CMF and FEC
