# Supplementary material for: Influence of chemotherapeutic drug-related gene polymorphisms on toxicity and survival of early breast cancer patients receiving adjuvant chemotherapy
Source: BMC Cancer. 2017 Jul 26;17:502. doi: 10.1186/s12885-017-3483-2 (PMC5530465; doi:10.1186/s12885-017-3483-2)
Supplement: Supplementary file 4 — Association among gene polymorphisms and risk of toxicity of any grade (grade1–2–3-4 vs 0). (DOC 83 kb) [file 12885_2017_3483_MOESM4_ESM.doc]

**Table S4. Association among gene polymorphisms and risk of toxicity of any grade (grade1-2-3-4 vs 0)**

|  | **HEMATOLOGIC TOXICITY** | | | | | | | | | | | | | | **NON-HEMATOLOGIC TOXICITY** | | | | | | | | | | | | | |
| --- | --- | --- | --- | --- | --- | --- | --- | --- | --- | --- | --- | --- | --- | --- | --- | --- | --- | --- | --- | --- | --- | --- | --- | --- | --- | --- | --- | --- |
|  | **LEUCOPENIA** | | | | | | | **NEUTROPENIA** | | | | | | | **STOMATITIS** | | | | | | | **HEPATIC** | | | | | | |
| **Genotype** | **0** | **1-2-3-4** | | | | **OR (95%CI) p** | | **0** | **1-2-3-4** | | | | **OR (95%CI)** | **p** | **0** | **1-2-3-4** | | | | **OR (95%CI)** | **p** | **0** | **1-2-3-4** | | | | **OR (95%CI)** | **p** |
|  |  |  |  |  |  |  |  |  |  |  |  |  |  |  |  |  |  |  |  |  |  |  |  |  |  |  |  |  |
| **GSTT1** |  | | | | | | |  | | | | | | |  | | | | | | |  | | | | | | |
| null | 40 | 10 | | | | 1 (reference)  1.32 (0.61-2.83) 0.483 | | 37 | 13 | | | | 1 (reference) | | 37 | 13 | | | | 1 (reference) | | 41 | 9 | | | | 1 (reference) | |
| present | 146 | 48 | | | |  |  | 144 | 50 | | | | 0.99 (0.49-2.01) 0.974 | | 161 | 33 | | | | 0.58 (0.28-1.22) 0.150 | | 168 | 26 | | | | 0.70 (0.31-1.62) 0.410 | |
| **GSTM1** |  | | | | | | |  | | | | | | |  | | | | | | |  | | | | | | |
| Null | 99 | 33 | | | | 1 (reference) | | 93 | 39 | | | | 1 (reference) | | 113 | 19 | | | | 1 (reference) | | 118 | 14 | | | | 1 (reference) | |
| Present | 87 | 25 | | | | 0.86 (0.48-1.56) 0.624 | | 88 | 24 | | | | 0.65 (0.36-1.17) 0.150 | | 85 | 27 | | | | 1.89 (0.98-3.62) 0.055 | | 91 | 21 | | | | 1.94 (0.94-4.03) 0.074 | |
| **GSTP1** |  | | | | | | |  | | | | | | |  | | | | | | |  | | | | | | |
| AA | 111 | 34 | | | | 1 (reference) | | 109 | 36 | | | | 1 (reference) | | 117 | 28 | | | | 1 (reference) | | 124 | 21 | | | | 1 (reference) | |
| AG | 71 | 23 | | | | 1.08 (0.59-1.98) 0.812 | | 68 | 26 | | | | 1.18 (0.66-2.12) 0.583 | | 77 | 17 | | | | 0.90 (0.46-1.74) 0.752 | | 81 | 13 | | | | 0.96 (0.46-2.03) 0.921 | |
| GG | 2 | 1 | | | |  | | 2 | 1 | | | |  | | 3 | 0 | | | |  | | 2 | 1 | | | |  | |
| **RCF1** |  | | | | | | |  | | | | | | |  | | | | | | |  | | | | | | |
| GG | 59 | 15 | | | | 1 (reference) | | 58 | 16 | | | | 1 (reference) | | 56 | 18 | | | | 1 (reference) | | 61 | 13 | | | | 1 (reference) | |
| GA | 82 | 31 | | | | 1.49 (0.74-3.00) 0.268 | | 78 | 35 | | | | 1.63 (0.82-3.22) 0.162 | | 97 | 16 | | | | 0.51 (0.24-1.09) 0.081 | | 100 | 13 | | | | 0.61 (0.26-1.40) 0.244 | |
| AA | 45 | 12 | | | | 1.05 (0.45-2.46) 0.913 | | 45 | 12 | | | | 0.97 (0.42-2.25) 0.937 | | 45 | 12 | | | | 0.83 (0.36-1.90) 0.659 | | 48 | 9 | | | | 0.88 (0.35-2.23) 0.787 | |
| AA vs. GA+GG |  |  |  |  |  | 0.82 (0.40-1.68) 0.582 | |  |  |  |  |  | 0.71 (0.35-1.45) 0.349 | |  |  |  |  |  | 1.20 (0.57-2.51) 0 .628 | |  |  |  |  |  | 1.16 (0.51-2.65) 0.722 | |
| **MTHFR** |  | | | | | | |  | | | | | | |  | | | | | | |  | | | | | | |
| CC | 53 | 12 | | | | 1 (reference) | | 51 | 16 | | | | 1 (reference) | | 54 | 12 | | | | 1 (reference) | | 61 | 6 | | | | 1 (reference) | |
| CT | 83 | 33 | | | | 1.82 (0.87-3.83) 0.114 | | 82 | 34 | | | | 1.32 (0.66-2.63) 0.428 | | 95 | 21 | | | | 0.92 (0.43-1.98) 0.828 | | 96 | 20 | | | | 2.12 (0.80-5.57) 0.128 | |
| TT | 48 | 13 | | | | 1.24 (0.52-2.98) 0.628 | | 48 | 13 | | | | 0.86 (0.38-1.98) 0.729 | | 49 | 12 | | | | 1.02 (0.42-2.44) 0.969 | | 52 | 9 | | | | 1.76 (0.59-5.27) 0.313 | |
| TT vs. CT+CC |  |  |  |  |  | 0.83 (0.41-1.67) 0.603 | |  |  |  |  |  | 0.72 (0.36-1.44) 0.354 | |  |  |  |  |  | 1.07 (0.52-2.23) 0 .850 | |  |  |  |  |  | 1.04 (0.46-2.37) 0.916 | |
| **TS-TR** |  | | | | | | |  | | | | | | |  | | | | | | |  | | | | | | |
| 2R/2R | 60 | 18 | | | | 1 (reference) | | 55 | 23 | | | | 1 (reference) | | 57 | 21 | | | | 1 (reference) | | 68 | 10 | | | | 1 (reference) | |
| 2R/3R | 66 | 20 | | | | 1.01 (0.49-2.09) 0.978 | | 68 | 18 | | | | 0.63 (0.31-1.29) 0.208 | | 76 | 10 | | | | 0.36 (0.16-.82) 0.015 | | 75 | 11 | | | | 1.00 (0.40-2.50) 0.995 | |
| 3R/3R | 60 | 20 | | | | 1.11 (0.54-2.31) 0.777 | | 58 | 22 | | | | 0.91 (0.45-1.81) 0.782 | | 65 | 15 | | | | 0.63 (0.30-1.33) 0.969 | | 66 | 14 | | | | 1.44 (0.60-3.48) 0.414 | |
| 3/3R vs. 2/3R+2/2R |  |  |  |  |  | 1.10 (0.59-2.06) 0.753 | |  |  |  |  |  | 1.14 (0.62-2.08) 0.675 | |  |  |  |  |  | 0.99 (0.50-1.96) 0.978 | |  |  |  |  |  | 1.44 (0.69-3.02) 0.328 | |

OR: Odds Ratio; CI: Confidence Interval.
